# SurvConvMixer: robust and interpretable cancer survival prediction based on ConvMixer using pathway-level gene expression images

**DOI:** 10.1186/s12859-024-05745-2

**Published:** 2024-03-27

**Authors:** Shuo Wang, Yuanning Liu, Hao Zhang, Zhen Liu

**Affiliations:** 1 College of Computer Science and Technology, Jilin University, Qianjin Street, Changchun, 130012 Jilin China; 2 Key Laboratory of Symbolic Computation and Knowledge Engineering of Ministry of Education, Jilin University, Qianjin Street, Changchun, 130012 Jilin China; 3 Graduate School of Engineering, Nagasaki Institute of Applied Science, Nagasaki, Japan

**Keywords:** Pathways in cancer, Gene expression data, Survival prediction, Robustness, Interpretable machine learning

## Abstract

Cancer is one of the leading causes of deaths worldwide. Survival analysis and prediction of cancer patients is of great significance for their precision medicine. The robustness and interpretability of the survival prediction models are important, where robustness tells whether a model has learned the knowledge, and interpretability means if a model can show human what it has learned. In this paper, we propose a robust and interpretable model SurvConvMixer, which uses pathways customized gene expression images and ConvMixer for cancer short-term, mid-term and long-term overall survival prediction. With ConvMixer, the representation of each pathway can be learned respectively. We show the robustness of our model by testing the trained model on absolutely untrained external datasets. The interpretability of SurvConvMixer depends on gradient-weighted class activation mapping (Grad-Cam), by which we can obtain the pathway-level activation heat map. Then wilcoxon rank-sum tests are conducted to obtain the statistically significant pathways, thereby revealing which pathways the model focuses on more. SurvConvMixer achieves remarkable performance on the short-term, mid-term and long-term overall survival of lung adenocarcinoma, lung squamous cell carcinoma and skin cutaneous melanoma, and the external validation tests show that SurvConvMixer can generalize to external datasets so that it is robust. Finally, we investigate the activation maps generated by Grad-Cam, after wilcoxon rank-sum test and Kaplan–Meier estimation, we find that some survival-related pathways play important role in SurvConvMixer.

## Introduction

Nowadays, cancer is still regarded as a challenging medical problem universally. In 2020, there were almost 10 million deaths caused by cancer. [1] In this era of precision medicine, cancer diagnosis and therapy are no longer limited to the one-size-fits-all approach, which treats patients through conventional treatment plans for their cancer types. With the help of high-throughput sequencing technology, cancer treatment strategies can be designed at the molecular level. And computational methods can be introduced for conducting analysis on the huge amounts of high-throughput sequencing data—including the survival prediction of cancer patients using their gene expression data.

In the early years, cancer survival prediction methods were mainly statistical models, such as Kaplan–Meier Estimation [2], Cox Proportional Hazard Regression [3], and so on. These statistical methods have been widely used by researchers. But they have a major shortcoming, namely, statistical methods only perform well on low dimensional data, such as the clinical data. More powerful methods are needed to mine the enormous knowledge behind the high dimensional data, such as the gene expression data. And then, the Machine Learning (ML) methods were used to make better use of high dimensional data. For instance, Support Vector Machines (SVM) were leveraged to select genes for cancer classification [4], and some researchers used Naive Bayes and K-Nearest-Neighbor (KNN) models trained on gene expression data for cancer prognosis prediction [5]. In addition, Artificial Neural Networks (ANN or simply, NN) have been used for cancer survival prediction through gene expression data, and displayed potential on heterogeneous data because they could be trained on gene expression and clinical data simultaneously [6].

Recent years have witnessed the explosive growth of hardware computation power. Nvidia’s GPUs with built-in CUDA cores and Google’s TPUs make training extremely huge Deep Learning (DL) models possible. Deep Learning models can learn the nonlinear relations between features, so that they have strong fitting capability. They also have tremendous potential for learning the relevance among high dimensional features. And naturally, Deep Learning models have been widely applied for cancer survival prediction using high dimensional data. For example, some researchers used Deep Learning model to predict cancer patients’ gene expression profile and studied their survival outcome by grouping them into different gene expression groups [7]. Some researchers integrated gene expression and clinical data into the Deep Learning model for better prediction of cancer overall survival [8]. Moreover, multi-modal Deep Learning model was used for cancer survival prediction, in which multi-omics data were trained simultaneously into a multi-input model, and achieved good performance [9].

Although Deep Learning models have great fitting capability, they are not perfect. One of their drawbacks is that they are data hungry, which means that, they usually need large amounts of data to train well. Shifting perspective on bioinformatics scenarios, the situation could be worse, especially when dealing with genomics data. Because the dimension of genomics data is very high, and significantly exceeds the sample size. So, overfitting is a non-negligible problem in Deep-Learning-based cancer survival prediction using genomics data. Many researchers used the average metrics values from k-fold cross-validation (CV) experiments to show their models’ performance, which we call internal validation (IV). However, that alone is not sufficient. The reason is that the train set and test set are from the same source. So they usually keep similar distribution. Although a model gets good metrics scores through internal validation, it may deliver bad results on an independent external dataset. This is called the batch effect [10]. In this paper, we define a model as robust if it can make effective predictions on an independent external dataset.

Another weakness of Deep Learning models is that they are hard to interpret. Due to the vast number of hidden nodes, it is difficult for us to interpret how a Deep Learning model predicts or what it has learned. However, when developing a cancer survival model, its interpretability deserves strong consideration. Since users usually prefer models that they believe they can at least partially understand.

In this paper, we aim at predicting cancer survival in a robust and interpretable way. Our main contributions are listed as follows:

- We proposed a novel gene expression data reformation scheme. In this process, we selected the genes in the KEGG Pathways in Cancer [11], and converted their expression values into two-dimensional (2D) gene expression patches. Then we concatenated these patches and finally get the pathway-level gene expression images.
- We leveraged the idea from ConvMixer [12] to build our model, namely, the SurvConvMixer. In the model, the learned representations were always at the pathway level throughout all hidden layers, which helped us understand the model.
- For each type of cancer, we selected an independent external dataset, which, despite being from a different platform than the training data, was used for external validation (EV). This allowed us to assess the robustness of the trained model.
- We tried to interpret the model’s prediction by Gradient-weighted Class Activation Mapping (Grad-Cam) [13]. Using the activation maps produced by Grad-Cam, we conducted wilcoxon rank-sum test to find the statistically significant pathways, thereby we could know which pathways the model paid more attention to. Finally, using Kaplan–Meier estimation, we tested whether these pathways were related to samples’ survival.

## Results

**Table 1:** Mean AUC scores of 50 times internal and external validation experiments.

	SurvConvMixer	SurvConvMixer Conv1D	KNN	SVM	Random Forest	Logistic Regression	Neural Network	GeneExpImgTL	PathCNN
*LUAD-Short-Term*									
IV	**0.6882**±**0.03**	0.6697 ± 0.03	0.5718 ± 0.09	0.5188 ± 0.10	0.5589 ± 0.08	0.5139 ± 0.10	0.4977 ± 0.12	0.6162 ± 0.09	0.6364 ± 0.03
EV	**0.6228** ± **0.08**	0.5570 ± 0.10	0.4899 ± 0.15	0.4709 ± 0.08	0.5415 ± 0.10	0.4982 ± 0.07	0.5379 ± 0.11	0.5109 ± 0.11	0.4519 ± 0.10
*LUAD-Mid-Term*									
IV	**0.6897** ± **0.03**	0.6670 ± 0.02	0.5167 ± 0.07	0.5426 ± 0.09	0.5603 ± 0.09	0.5223 ± 0.08	0.5371 ± 0.09	0.5779 ± 0.12	0.6618 ± 0.03
EV	**0.6291** ± **0.05**	0.5987 ± 0.04	0.5564 ± 0.03	0.5240 ± 0.04	0.5034 ± 0.06	0.5046 ± 0.04	0.5291 ± 0.05	0.5322 ± 0.08	0.5249 ± 0.03
*LUAD-Long-Term*									
IV	0.7095 ± 0.02	**0.7185** ± **0.03**	0.5282 ± 0.12	0.6209 ± 0.12	0.6163 ± 0.11	0.6623 ± 0.13	0.6705 ± 0.13	0.5536 ± 0.09	0.6826 ± 0.02
EV	**0.6272** ± **0.05**	0.5224 ± 0.06	0.5812 ± 0.04	0.5659 ± 0.04	0.5229 ± 0.07	0.5390 ± 0.04	0.5140 ± 0.06	0.5115 ± 0.13	0.5510 ± 0.05
*LUSC-Mid-Term*									
IV	0.5751 ± 0.07	0.6099 ± 0.08	0.5147 ± 0.08	0.5669 ± 0.08	0.6008 ± 0.07	0.5346 ± 0.08	0.5622 ± 0.08	0.5429 ± 0.11	**0.6612** ± **0.08**
EV	0.5597 ± 0.06	0.5350 ± 0.06	0.4843 ± 0.04	0.4971 ± 0.04	0.4619 ± 0.07	0.5180 ± 0.04	0.4965 ± 0.05	0.4885 ± 0.06	**0.5673** ± **0.08**
*LUSC-Long-Term*									
IV	0.6081 ± 0.09	**0.6327** ± **0.09**	0.5114 ± 0.09	0.5842 ± 0.10	0.4963 ± 0.10	0.5621 ± 0.10	0.5460 ± 0.11	0.5403 ± 0.09	0.6143 ± 0.09
EV	**0.5893** ± **0.06**	0.5401 ± 0.05	0.5734 ± 0.05	0.5653 ± 0.03	0.5115 ± 0.06	0.4970 ± 0.04	0.5068 ± 0.05	0.5006 ± 0.03	0.4760 ± 0.07
*SKCM-Short-Term*									
IV	0.5933 ± 0.13	0.5537 ± 0.14	0.5659 ± 0.12	0.5917 ± 0.15	0.5665 ± 0.15	0.5722 ± 0.13	0.5716 ± 0.14	0.5335 ± 0.11	**0.6430** ± **0.12**
EV	**0.5628** ± **0.08**	0.5548 ± 0.09	0.4320 ± 0.07	0.4047 ± 0.04	0.5076 ± 0.08	0.4941 ± 0.06	0.5167 ± 0.07	0.4966 ± 0.05	0.4885 ± 0.10
*SKCM-Mid-Term*									
IV	0.6515 ± 0.08	0.6184 ± 0.06	0.5875 ± 0.06	0.6127 ± 0.06	0.6682 ± 0.06	0.5952 ± 0.06	0.5951 ± 0.06	0.6137 ± 0.05	**0.6912** ± **0.05**
EV	**0.6409** ± **0.11**	0.5726 ± 0.14	0.3266 ± 0.09	0.4141 ± 0.06	0.5240 ± 0.12	0.5166 ± 0.07	0.4197 ± 0.08	0.4073 ± 0.04	0.6087 ± 0.07

The bolded value indicates that this value is the best performance among all the models

### Samples Involved in the Experiments

First of all, we define three overall survival prediction problems as follows:

- *Short term overall survival prediction problem* to predict whether a sample survives after 1 year.
- *Mid term overall survival prediction problem* to predict whether a sample survives after 3 years.
- *Long term overall survival prediction problem* to predict whether a sample survives after 5 years.

The cancer overall survival (OS) prediction problems in this paper are to predict whether samples can survive beyond the selected survival time. Thus, we had to remove some samples because we could not label them. For example, if we want to predict the long-term OS of LUAD, and there is a sample whose OS state is survival and has an OS time of four years, we cannot label this kind of sample. So the sample size we use would change with the selected survival time. For LUAD, the sample size of train set and external validation set were 2210 versus 166 for short-term OS prediction, 1801 versus 121 for mid-term OS prediction, and 1499 versus 88 for long-term OS prediction. For LUSC, the sample size of train set and external validation set were 221 versus 104 for mid-term OS prediction, and 187 versus 99 for long-term OS prediction. For SKCM, the sample size of train set and external validation set were 426 versus 40 for short-term OS prediction, 344 versus 33 for mid-term OS prediction.

We did not implement short-term OS prediction for LUSC, because all samples in the external validation set of LUSC survived after one year. And we also did not conduct long-term OS prediction for SKCM due to all the samples in SKCM’s external validation set did not survive after five years.

### Performance of SurvConvMixer

**Fig. 1:**
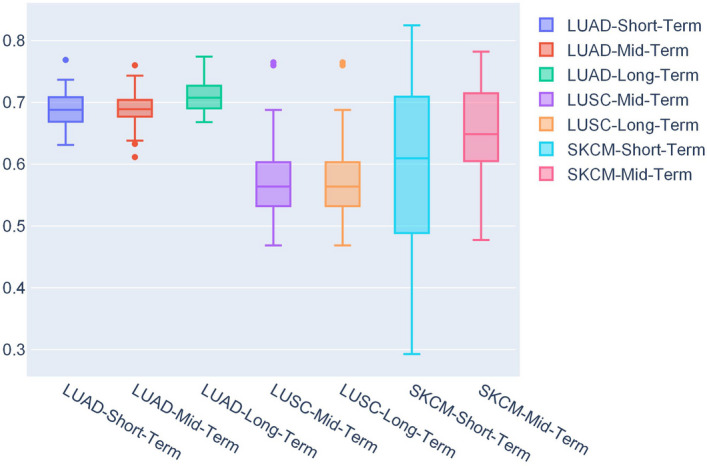
The AUC distributions of SurvConvMixer for internal validation

The average IV AUC values for all the prediction problems are listed in Table 1 with the tag IV. For different prediction problems, we can find that SurvConvMixer achieved 0.6882, 0.6897 and 0.7095 of average IV AUC scores on the three OS prediction problems of LUAD, respectively, all far greater than other OS prediction problems with SurvConvMixer. This may be because the LUAD train set’s sample size was much larger than the other two cancers, thereby the model could be trained more fully. Similar phenomenon happens when we compare the mid-term OS prediction problems between SKCM and LUSC, where the sample size of SKCM was 344, larger than LUSC’s 221. And the average IV AUC of SKCM mid-term OS was 0.6515, much greater than LUSC’s 0.5751. Figure 1 shows the distributions of IV AUC scores of SurvConvMixer. We can observe that, SurvConvMixer performed the best on LUAD, and the three IV AUC distributions of LUAD were more compact than others.

**Fig. 2:**
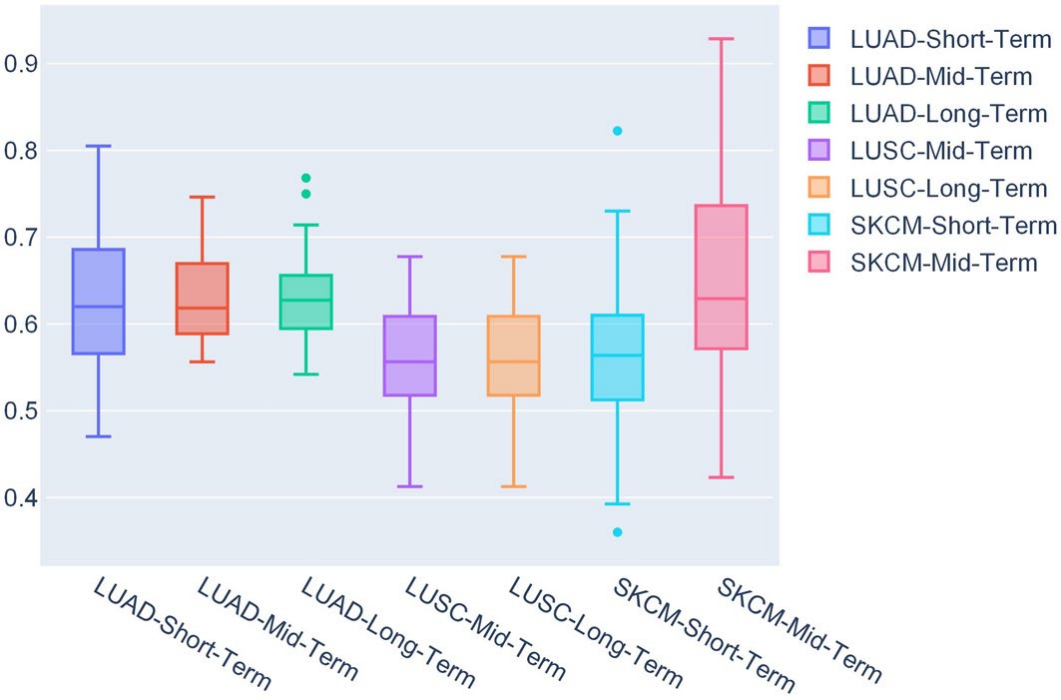
The AUC distributions of SurvConvMixer for external validation

The average EV AUC scores for all the prediction problems are listed in Table 1 with the tag EV. SurvConvMixer showed its strong generalization ability, it achieved greater-than $-$0.6 EV AUC scores in 4/7 of the OS prediction problems. In all the three OS prediction problems of LUAD, our model achieved greater-than $-$0.6 EV AUC scores, which were 0.6228, 0.6291 and 0.6272. In the SKCM mid-term OS prediction, we surprisingly observe that although the sample size of SKCM’s train data is fairly small, SurvConvMixer generalized well in its EV data. Such results illustrated that SurvConvMixer had the capability to generalize, thereby it was robust. Figure 2 shows the distributions of EV AUC scores of SurvConvMixer. We can also see that the EV AUC distributions of LUAD were more compact. And all of its EV AUC scores had median values larger than 0.5. And in Fig. 5, we can find that the SKCM mid-term train data had a very different label distribution compared to its EV data. And our model was very robust on prediction of SKCM’s mid-term OS because it could even generalize to external data with such striking differences in distribution.

### Comparison with benchmark methods

#### Introduction of benchmark methods

The benchmark methods we selected in this paper were: SurvConvMixerConv1D, K-Nearest Neighbors algorithm (KNN), Support Vector Machine (SVM), Random Forest, Logistic Regression, Neural Network, GeneExpImgTL and PathCNN. In this paper, we conducted comparison experiments using the 276 genes in Kegg Pathways in Cancer except GeneExpImgTL, because GeneExpTmgTL needed much more genes to construct its gene expression images. In this paper, we ran the five Machine Learning methods using scikit-learn package [14]. And grid search [15] was used to search for their best hyper-parameters. First of all, the introduction and hyper-parameter settings of these benchmark methods are listed as follows:

- *SurvConvMixerConv1D*: It is Another version of SurvConvMixer. The only difference was that in SurvConvMixerConv1D, all convolution kernels were set to be one-dimensional. Our aim was to validate the effectiveness of two-dimensional convolution kernels, thereby demonstrating that the spatial information we added to gene expression data was meaningful.
- *KNN*: K-Nearest Neighbors algorithm is a kind of Machine Learning method. The KNN classifier assigns unlabeled observations to the class of the most closely related labeled examples, thus facilitating their classification [16]. In this paper, we obtained leaf_size = 10, n_neighbors = 2 and euclidean distance (namely,* p* = 2) as the hyper-parameters of KNN.
- *SVM*: Support Vector Machine classifies data by finding the optimal hyperplane to separate different categories [15]. In this paper, we obtained C = 10, gamma = 0.1 and rbf kernel as its hyper-parameters.
- *Random forest*: Random Forest classifies by combining decisions from multiple decision trees, ensuring high accuracy and robustness [17]. In this paper, n_estimators = 250, min_samples_split = 2, min_samples_leaf = 1, max_features = sqrt and max_depth = 15 were set as its hyper-parameters.
- *Logistic regression*: Logistic regression predicts class probabilities using a logistic function to classify data [18]. In this paper, C = 10 and penalty = l2 were obtained as its hyper-parameters.
- *Neural network*: Neural networks classify data by learning patterns through training and applying them to new inputs [6]. In this paper, rectified linear unit was selected as the activation function of Neural Network, other hyper-parameters were: alpha = 0.0001, hidden_layer_sizes = (32, 144, 32), learning_rate = constant and solver = adam.
- *GeneExpImgTL*: GeneExpImgTL was a lung cancer survival prediction method which leveraged KEGG BRITE hierarchical data and R package Treemap to structuralize samples’ gene expression data into image. Then the CNN model was used to predict lung cancer survival (270 days progression free survival in their paper) [19]. We firstly selected 1000 salient genes by mutual information selector. Then gene expression images with size 27*27 were generated according to their method. After Keras Tuning, a CNN model with two conv2d layers with 3*3 kernel and 128 filters was built to conduct comparison experiments, because their work also conducted hyper-parameter searching.
- *PathCNN*: PathCNN fused three omics data into images for glioblastoma survival prediction via CNN and Grad-Cam [20]. In this paper, we generated gene expression images using the 40 pathways in Kegg Pathways in Cancer. And 5 principal components were obtained for each pathway, which was the same as PathCNN. Namely, we generated images with size 5*40. Finally, a same CNN model with same hyper-parameters with PathCNN was built for comparison experiments.

#### Internal validation (IV)

**Fig. 3:**
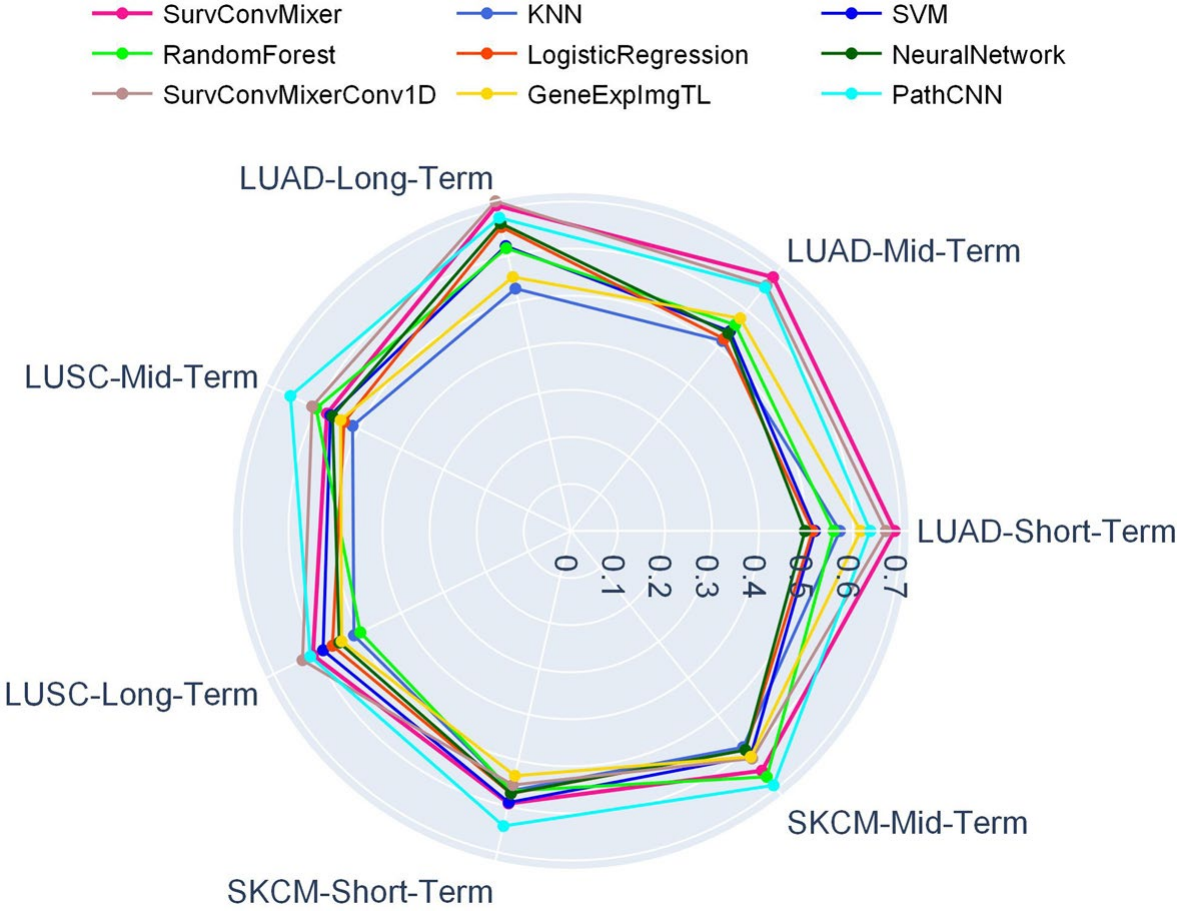
Comparison of SurvConvMixer and benchmark methods for internal validation

In most datasets and selected survival terms, the SurvConvMixer and SurvConvMixerConv1D achieved mean AUC scores which were higher or comparable to other benchmark methods. As shown in Table 1 with the tag IV, we can observe that SurvConvMixer achieved a mean AUC of 0.6882 in LUAD-Short-Term OS prediction, 0.6897 in LUAD-Mid-Term OS prediction. And SurvConvMixerConv1D achieved 0.7185 in LUAD-Long-Term OS prediction, and 0.6327 in LUSC-Long-Term OS prediction. These four scores were the best among all the models. And our models’ strong performance on LUAD indicated that, when the datasets get larger, the SurvConvMixer models have better fitting capabilities on training data than other benchmark methods. Because the dataset of LUAD had much more samples than LUSC and SKCM. On the other hand, PathCNN was better at smaller datasets. Because PathCNN achieved 0.6612 in LUSC-Mid-Term OS prediction, 0.6430 in SKCM-Short-Term OS prediction and 0.6912 in SKCM-Mid-Term OS prediction, which were the best among all the models. We visualize the average IV AUC scores of SurvConvMixer and the benchmark models as a radar chart in Fig. 3. In the radar chart, we can easily observe that SurvConvMixer was much better than other benchmark methods in LUAD, and PathCNN was better in SKCM.

#### External validation (EV)

**Fig. 4:**
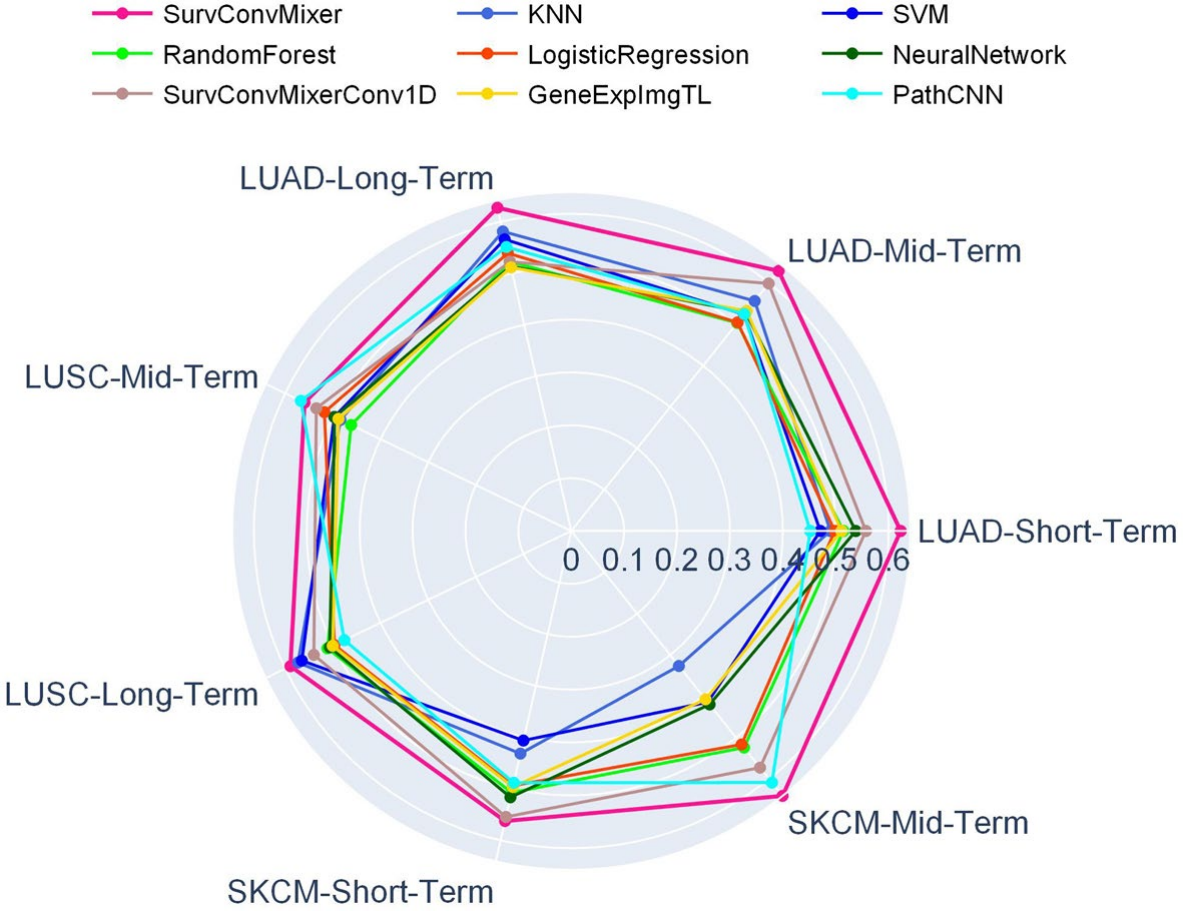
Comparison of SurvConvMixer and benchmark methods for external validation

Internal validation (IV) can test the train set fitting ability of models. However, through IV, it is hard to test whether a model is reliable. Because the data which are independent from the train set usually have different distributions. So external validation (EV) is necessary to test the generalization capabilities of models. As shown in Table 1 with the tag EV, we can observe that among all the seven target problems, SurvConvMixer achieved the highest AUC scores on six of them, except for LUSC-Mid-Term OS prediction. What deserves special attention is that, SurvConvMixer achieved 0.6228 in LUAD-Short-Term OS prediction, 0.6291 in LUAD-Mid-Term OS prediction, 0.6272 in LUAD-Long-Term OS prediction and 0.6409 in SKCM-Long-Term OS prediction. These four AUC scores were significantly higher than other benchmark models. The adavantages of SurvConvMixer became more evident in EV. This indicated that SurvConvMixer had better generalization capabilities and could better handle unseen data. We also visualize the average EV AUC scores of SurvConvMixer and the benchmark models as a radar chart in Fig. 4. In this radar chart, the graph of SurvConvMixer enclosed nearly all the radar graphs of other benchmark methods, which illustrated that SurvConvMixer was the most robust model compared to other benchmark methods. What’s more, in Table 1, we can observe that SurvConvMixer exhibited relatively small performance differences between internal and external validation. This suggested that the model was less sensitive to distributional differences between training and testing data, making it more robust.

#### Advantages of Two-Dimensional Convolution Kernel

In Table 1, we can observe that compared to SurvConvMixerConv1D model, SurvConvMixer generally performed better in most cases. What deserves special attention is that in LUAD-Long-Term OS prediction, SurvConvMixerConv1D achieved 0.7185 IV AUC score, greater than SurvConvMixer’s 0.7095. But SurvConvMixerConv1D achieved only 0.5224 EV AUC score, far below SurvConvMixer’s 0.6272. These comparisons between SurvConvMixer and SurvConvMixerConv1D indicated that two-dimensional convolution kernels were better at extracting more useful features than one-dimensional kernels on the pathway-level gene expression images we created. One of the reasons is that for each sample we formatted its pathways’ gene expression values into two-dimensional patches, and then we concatenated these patches into gene expression image. Thus we gave the gene expression data spatial information, and the the differences between the pathways became more significant. But the one-dimensional convolution kernels were not good at extracting spatial information. In the Methods section, we will introduce the generation process of the pathway-level gene expression images in detail.

**Fig. 5:**
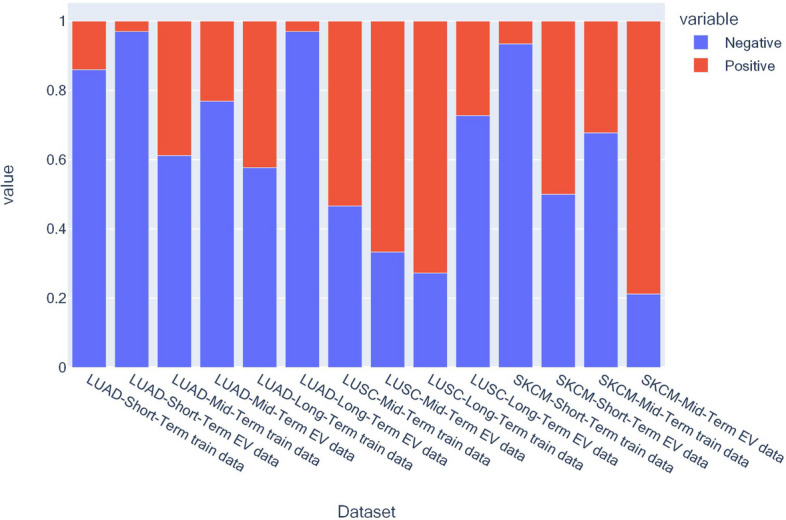
Sample label distributions of short-term, mid-term and long-term overall survival predictions

**Fig. 6:**
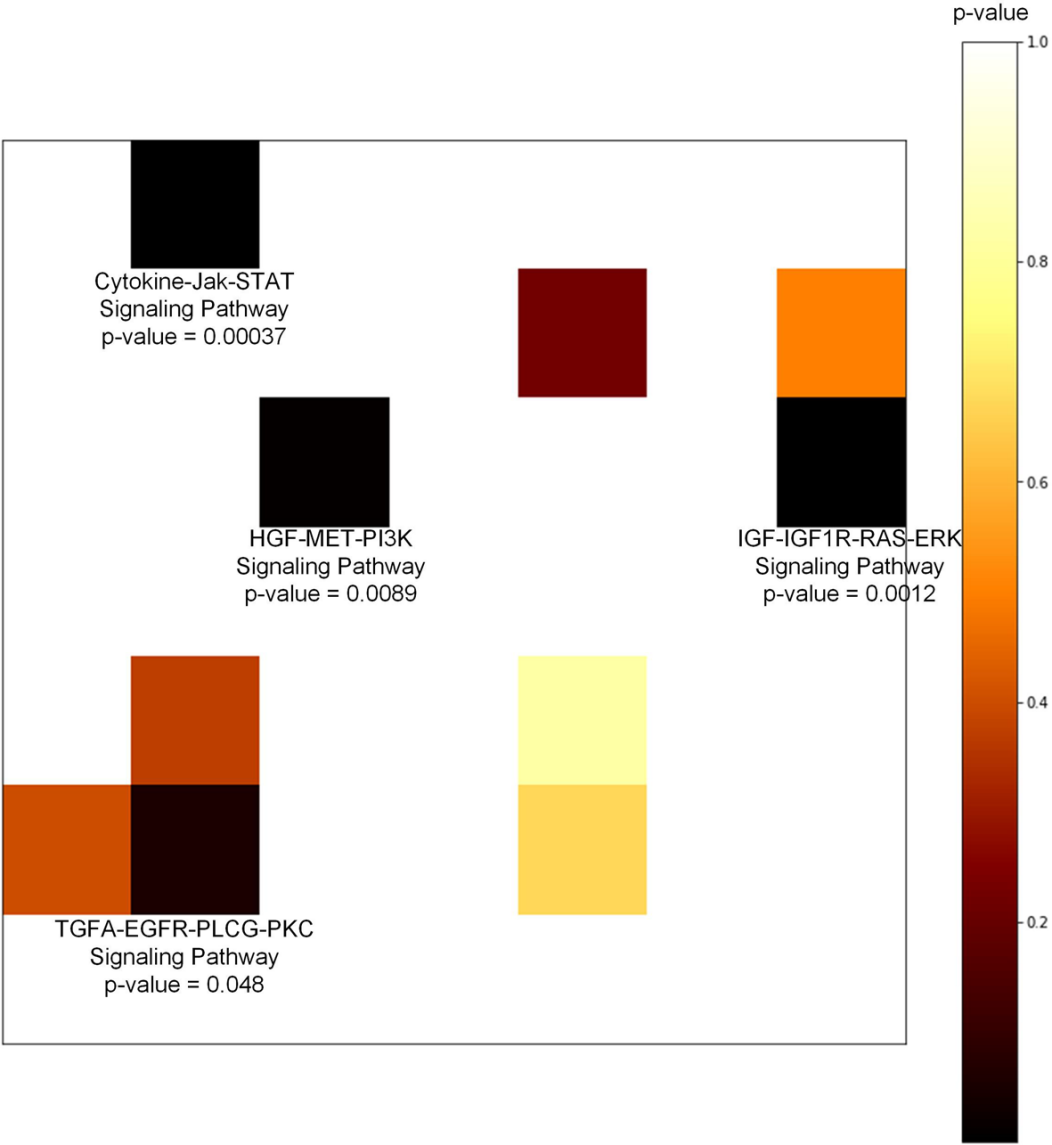
The visualization of Wilcoxon sum-rank test pathway-by-pathway of SKCM mid-term overall survival prediction. The color of patches indicate p values of pathways, the darker means the more significant

**Fig. 7:**
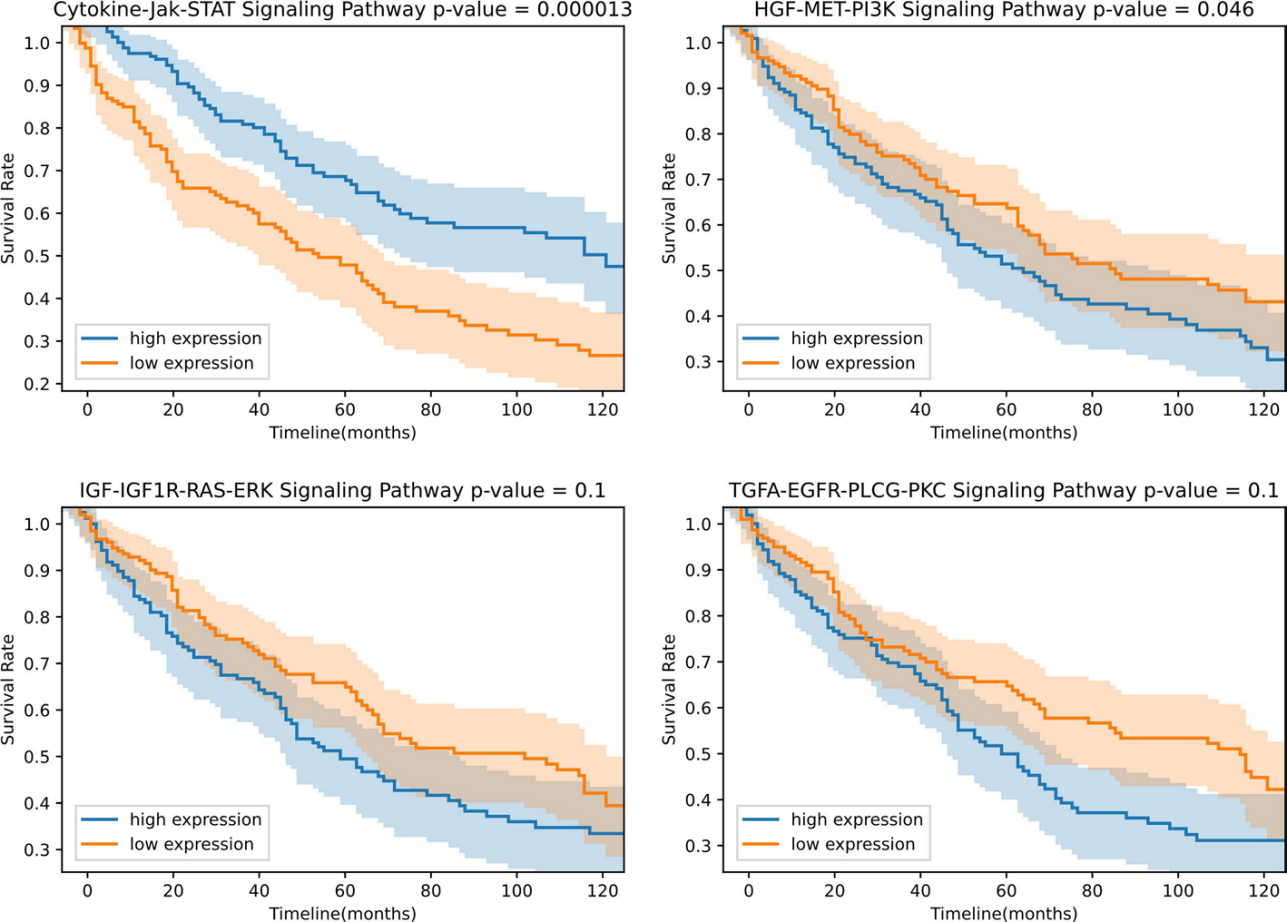
Kaplan–Meier survival curves for the four key pathways found by Wilcoxon sum-rank test of SKCM samples

### Key pathways finding

We conducted the key pathways finding process on all the seven OS prediction problems. The detailed process of key pathways finding will be introduced in the Methods section. However, it did not work for LUAD and LUSC. Because nearly all of the 40 pathways were not statistically significant in terms of the survival of LUAD and LUSC. But things changed when we applied the process on SKCM. We tried to find key pathways in SKCM mid-term OS prediction due to its great EV performance. The results of Wilcoxon test are shown in Fig. 6, where four pathways with p value less than 0.05 were found. They were Cytokine-Jak-STAT signaling pathway, HGF-MET-PI3K signaling pathway, IGF-IGF1R-RAS-ERK signaling pathway and TGFA-EGFR-PLGG-PKC signaling pathway. Then Kaplan–Meier estimations were implemented by the median GSVA pathway expression values of these four pathways. In Fig. 7, with the log-rank test p values, we can observe that the Cytokine-Jak-STAT signaling pathway and HGF-MET-PI3K signaling pathway were significantly relevant to the survival of TCGA SKCM samples, whereas IGF-IGF1R-RAS-ERK signaling pathway and TGFA-EGFR-PLGG-PKC signaling pathway were not significant enough (p value larger than 0.05).

## Methods

**Fig. 8:**
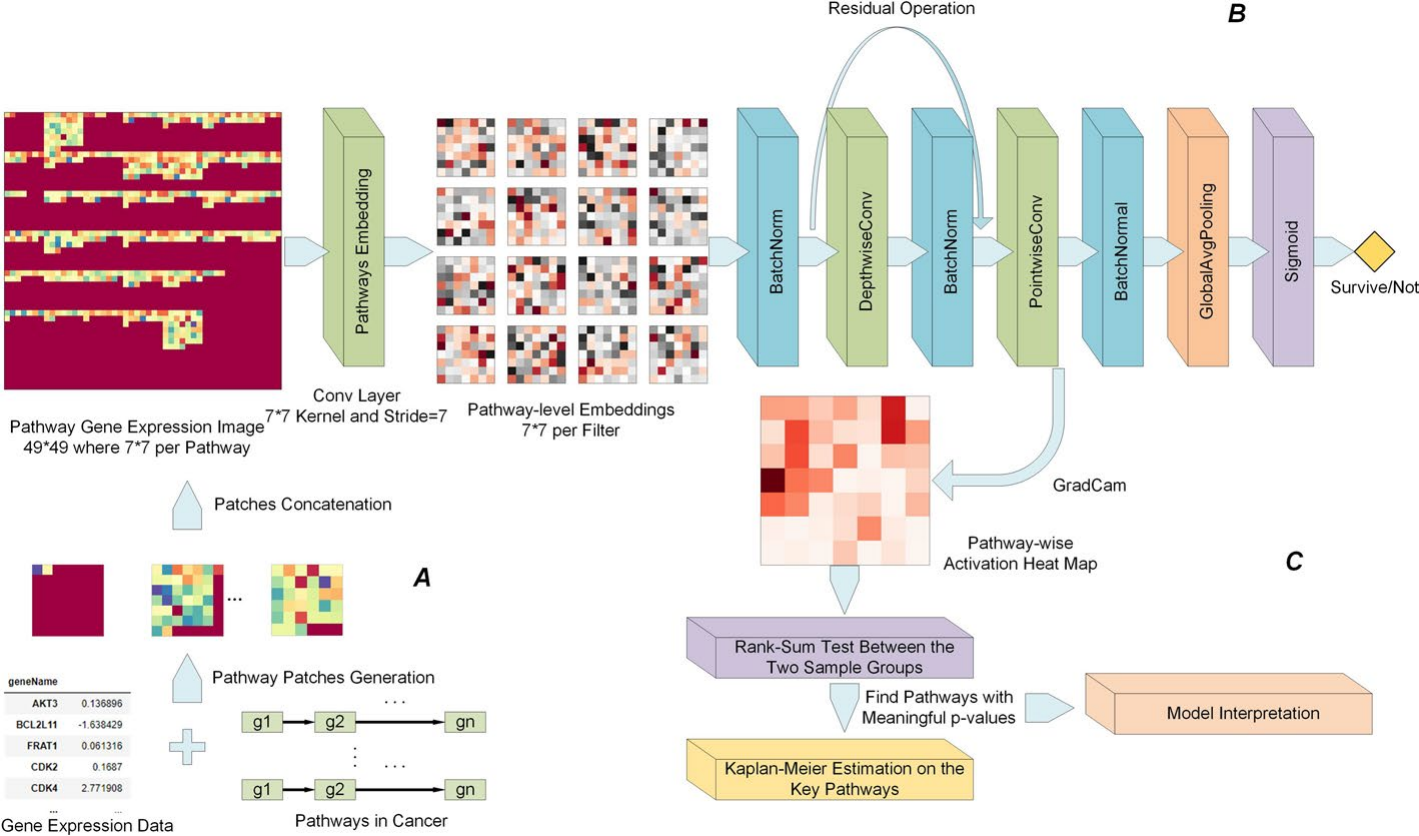
The entire workflow of the SurvConvMixer. A: Pathway-wise gene expression image generation. B: The overall architecture of SurvConvMixer. C: Key pathways finding and model interpretation

**Table 2:** Datasets used in the paper

	Train Data	External Validation Data
LUAD	TCGA-LUAD, GSE2088, GSE2514, GSE5843, GSE10072, GSE11969, GSE12667, GSE13213, GSE17475, GSE20853, GSE20875, GSE26939, GSE29016, GSE31210, GSE32863, GSE41271, GSE72094, GSE68571, GSE83227, GSE68465	Oncosg_2020
LUSC	TCGA-LUSC	GSE74777
SKCM	TCGA-SKCM	Dfci_2015

### Data

The gene expression data we selected in this paper are listed in Table 2. For LUAD, we used both the TCGA [21] and GEO [22] data to train the model. Subsequently, an independent external dataset was downloaded from the cBioPortal database [23] for testing the robustness of the model. For LUSC, we trained the model on the TCGA dataset, and performed external validation on a GEO dataset. For SKCM, its TCGA dataset was also selected as train set, because it was larger. And a dataset from cBioPortal was selected as an external validation set.

### Pathway level gene expression image generation

#### Gene expression data pre-processing

The data we used in this paper are from different platforms. So they had already been pre-processed in different manners. For all of these datasets, we renormalized them with Min-Max normalization, which can be summarized as the following:

1 $$\begin{aligned} X_{scaled} = \frac{X - X_{min}}{X_{min}X_{max}} \end{aligned}$$

where *X* denotes the expression values of a gene over all samples, $X_{min}$ and $X_{max}$ denote this gene’s minimum and maximum expression values. Although after renormalization, these datasets may have significantly different distributions, we can leverage this characteristic to test whether our model can learn genuine biological knowledge, or can merely learn the distribution bias.

#### Gene expression data structuralization

A novel gene expression data structuralization method is proposed. The process of this method is shown in Fig. 8A. Firstly, we downloaded the pathways in KEGG Pathways in Cancer. This collection contains 40 pathways related to 276 genes. Then we extracted the expression data of these genes. For each pathway, its expression vectors were generated for all the samples. If there were missing gene expression values for some samples, we padded zeros into the corresponding positions in the pathway gene expression vectors to maintain the same shape of each pathway among all samples. We next converted the pathway gene expression vectors into 2D patches.The orders of the genes in a patch were the same as their orders in the pathway. Because the longest pathway in KEGG Pathways in Cancer contains 46 genes, we padded zeros behind each pathway vector to let each pathway vector have the same length of 49. Then these vectors were reshaped into 7*7 patches. For each sample, we added nine 7*7 patches with zero values at the end of the pathway patches. Finally, for each sample, we concatenated its patches into a 49*49 image. That is to say, if the entire image was regarded as consisting of 7*7 patches, then each of the first 40 patches represented a pathway, while the last two patches in the second-to-last row and all 7 patches in the last row were filled with zero values. This shape was designed to facilitate the model’s patch-wise processing. Additionally, SurvConvMixer skipped these added blank patches during training, ensuring that these patches with zero values did not affect the calculation of the model’s loss function.

The main reason for structuralizing one-dimensional gene expression data into two-dimensional images is to utilize the powerful feature extraction capabilities of computer vision models, especially Convolutional Neural Networks (CNNs) [24]. And the generated 2D matrices essentially serve as a form of data augmentation or re-representation. It is worth noting that inside each pathway gene expression patch, we let the padded zeros take part in the training of our model, which was different from the zero patches added at the end of each image. This is because with these padded zero values inside each patch, the gene expression of each pathway obtained extra spatial information, which helped the model to reveal underlying structures and patterns in the data.

### Sample labeling

In this paper, we formulate the cancer survival prediction as a problem to predict whether a sample survives after the selected time, which is actually a classification task. So we labeled the samples according to their survival status and the selected survival time. The labeling process can be summarized in Algorithm 1. This algorithm outlines a process for assigning labels to samples based on their survival outcomes, considering both the selected survival time and the overall survival time of each sample. Samples that survived beyond the selected time were labeled as 0, while those that experienced the event of death were labeled as 1. If a sample did not meet either of these criteria, it was removed from the dataset.

**Algorithm 1:**
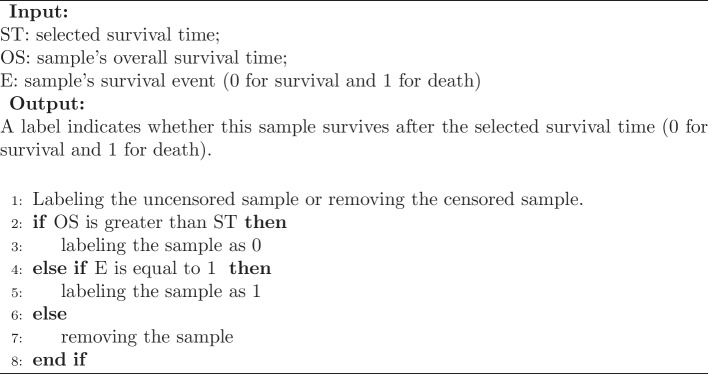
Sample Labeling Scheme

### SurvConvMixer model construction

The ConvMixer [12] was a kind of CNN model, which borrowed the embedding strategy of vision transformer (ViT) [25]. That is to say, ConvMixer embedded the input image into patches. In addition, ConvMixer learned the feature representations at the patch level throughout all the hidden layers. ConvMixer had much smaller parameter size than ViT, so it could be used on fairly small datasets without worrying about overfitting. In this paper, we built our prediction model based on ConvMixer, and we call it SurvConvMixer. The architecture of SurvConvMixer is shown in Fig. 8B. Firstly, we designed a convolution layer with 7*7 kernel and with a stride of 7, to embed the pathways gene expression image into 7*7 pathway level embeddings. Then a batch normalization layer was added to prevent gradient vanishing. The normalized embeddings were then sent to the depth-wise convolution layer (kernel size = 3 * 3, stride = 2) with residual connection to learn the representation per embedding. And a point-wise convolution layer was used to fuse the learned representations across all the channels. This combination of a depth-wise convolution layer and a point-wise convolution layer was called a ConvMixer block. In SurvConvMixer model, we repeated this block twice. It is worth noting that in the ConvMixer blocks, we set the padding as same to maintain the 7*7 shape of feature embeddings, which facilitates the subsequent model interpretation. Finally, a global average pooling layer and sigmoid activation were added for the final survival prediction. All the convolution layers we use have 256 filters. What’s more, a masking process was added before the prediction layer to mask the representations in the last two patches in the second-to-last row and all seven patches in the last row into zeros. Thus the SurvConvMixer model would skip these positions when calculating losses.

### Model validation scheme

To gain robust models, we introduced external datasets for external validation (EV). And internal validation (IV) was also applied for comparison. We used 5-fold cross-validation (CV) as IV. In each validation fold, 80% samples of the train set were selected for training and 20% for validation. And we let the model make predictions on the absolutely untrained external dataset before the end of each fold. We performed the 5-fold CV for 10 repeats, and evaluated the prediction performance based on the area under the curve (AUC) score.

### Key pathways finding by Grad-Cam

**Fig. 9:**
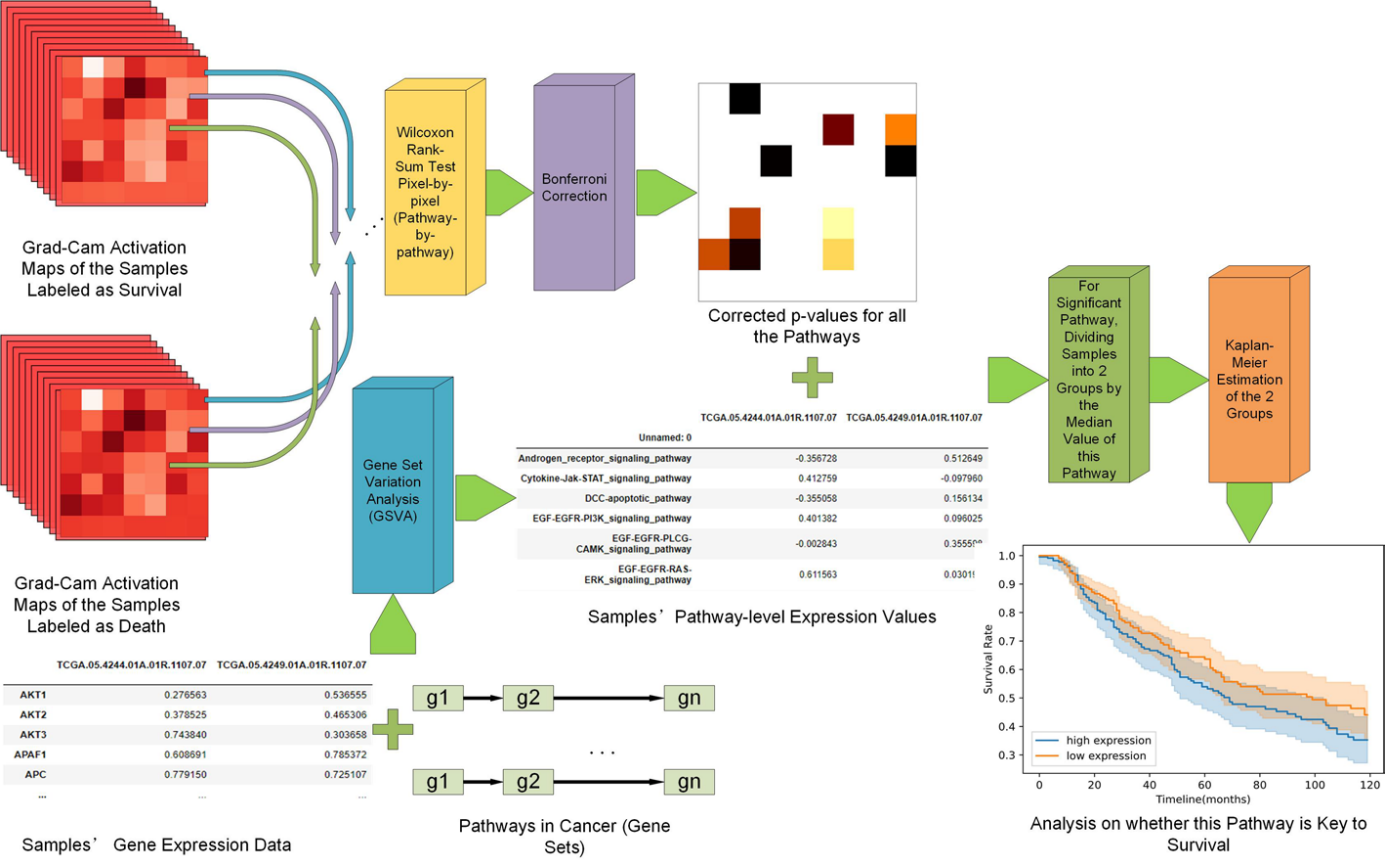
The detailed process of model interpretation, namely, the key pathways finding

The process of model interpretation by looking for key pathways are illustrated in Figs. 8C and 9, which contains: (1) Generating activation maps through Grad-Cam; (2) Wilcoxon rank-sum test between the two classes of samples to find key pathways; (3) Kaplan–Meier estimation on different expression level of key pathways.

#### Generating activation maps through Grad-Cam

Generally speaking, Deep Learning models such as CNNs are black boxes, which means we can not understand them by looking at one of their hidden nodes. But in some scenarios such as medical informatics, it is important for us to know why a model gives a specific prediction. And Grad-Cam, can show us the heat maps over the layers in the CNN model. It uses gradient-based localization to display what a model emphasizes on the learned 2D features. For a specific convolution layer and data from class c, we have n feature maps $A^{n}$. Then the importance of feature maps of class c can be calculated as follows:

2 $$\begin{aligned} \alpha _{n}^c = \overbrace{\frac{1}{Z}\sum _i\sum _j}^{Global Average Pooling}\cdot \underbrace{\frac{\delta y^c}{\delta A_{ij}^{n}}}_{Gradients Via Backprop} \end{aligned}$$

Where $y^c$ is the gradient scores before the sigmoid activation layer, and Z is the number of representations in a single feature map, in our model, it is 49. Then we can calculate the forward activation map of class c by combining the importance of feature maps and the learned representations of feature maps. And ReLU function will be used to filter out those below-zero values. The following equation shows how it works:

3 $$\begin{aligned} GradCam=ReLU\underbrace{(\sum _n\alpha _{n}^cA^{n})}_{Linear Combination} \end{aligned}$$

#### Wilcoxon rank-sum test between the two classes of samples

After having generated all the activation maps, we divided them into two groups by their ground truth label, namely, whether the samples survive the selected time or not. Then, we performed Wilcoxon rank-sum test among the two sets of samples per pathway. In other words, we conducted the test between the two groups on their activation maps patch-by-patch, except the last two zero patches in the second-to-last row and all seven zero patches in the last row. Then we obtained the p values for all the 40 pathways. We leveraged Bonferroni correction to correct these *p* values and obtained key pathways (pathways that had statistically significant p-values, namely *p* $< 0.05$).

#### Kaplan–Meier estimation on the key pathways

Firstly, we calculated the pathway level expression values for the 40 pathways by gene set variation analysis (GSVA) [26]. It could measure the variation of pathway activity across all the samples by the gene expression values inside the pathway. Through GSVA, we converted samples’ gene expression values into pathway expression values. Then, we regrouped all the samples by the median expression value of each key pathway. Finally, we conducted Kaplan–Meier estimation between the high expression group and low expression group and used log-rank test to calculate the* p* value of survival difference.

## Discussion

Cancer is one of the leading causes of death around the world. Survival analysis and prediction of cancer patients is of great significance for precision medicine in treating cancer patients. The robustness and interpretability of the survival prediction models are important, where robustness tells whether a model really has learned the knowledge, and interpretability means if a model is able to show human what it has learned. In this paper, we propose a robust and interpretable model SurvConvMixer, which leveraged pathways customized gene expression images and ConvMixer for cancer short-term, mid-term and long-term OS prediction. The remarkable performance of SurvConvMixer illustrated that the pathway images were biologically meaningful.

As stated earlier, external validation (EV) is necessary to prove models’ robustness. In this paper, we look for EV data for the three cancers which are independent from the train data. That is to say, the train data and EV data are from different platform. For example, the train data of LUAD are from TCGA and GEO, but the EV data does not belong to any of these two platforms. Our method achieved excellent results on the EV of LUAD and SKCM, which showed that SurvConvMixer was robust, and had strong generalization capability. By comparison between IV and EV of the same OS prediction problem, we can conclude that the IV-only model evaluation is insufficient. For example, in Table 1, PathCNN achieved a good IV AUC score on LUAD mid-term OS prediction of 0.6618. But its AUC score on EV was only 0.5249, which exhibited signs of overfitting.

Figure 5 shows us the label distributions for different OS prediction problems. We can observe that, for most of the prediction problems, their train dataset and EV dataset had similar distributions of positive and negative labels. But there were two exceptions, the SKCM-Mid-Term OS prediction and the LUSC-Long-Term OS prediction, both had exactly the opposite label distributions between the train dataset and the EV dataset. But they still achieved EV AUC scores of 0.6409 and 0.5893, which were notable performance, especially for SKCM-Mid-Term OS prediction, indicating that our model could learn the biological knowledge from the structured gene expression data.

In the key pathways finding subsection, we find that nearly all of the 40 pathways were not significantly relevant to survival for LUAD. But SurvConvMixer still achieved decent IV and EV performance on OS prediction problems of LUAD. This may be because, though the KEGG Pathways in Cancer are important for cancers, they may highly express in nearly all LUAD samples. So we could not find their statistical significance in terms of survival based on their median expression level. But it did not prevent SurvConvMixer from learning the non-linear relations among them, and our method still achieved good and generalized prediction performance.

## Conclusion

In this paper, we have introduced SurvConvMixer, a ConvMixer-based Deep Learning model leveraging pathway-wise gene expression images for cancer survival prediction. We have shown that our model outperformed other benchmark methods, especially in external validation experiments. And we have proved that SurvConvMixer was robust based on external validation. Finally, we show that with Grad-Cam and wilcoxon rank-sum test, our model became interpretable, and key pathways highly relevant to survival can be found. SurvConvMixer demonstrates the great prospects of using structured genomics data with novel Deep Learning models in the field of bioinformatics. In the future, we will further study ways to structuralize genomics data to make them more biologically meaningful. For example, the combination of pathway data and graph structure holds promise.

## Related works

### Applications of CNNs on genomics data

Convolutional Neural Networks (CNNs) are kind of variants of Deep Learning which are designed specifically for two-dimensional data. In the last few years, CNNs have made great achievements in the field of computer vision, such as image classification, image semantic segmentation, image generation, etc [27].

Genomics data, such as gene expression data, are usually small in sample size, but large in feature dimensionality. Conventional deep neural networks with fully connected layers tend to overfit on this kind of data. Since CNNs can extract high level features from the data, they can be used on gene expression data which has been converted to 2D gene expression matrices. Lopez et al. developed a CNN model for lung cancer survival prediction. They converted the gene expression data into gene expression images according to genes’ assigned categories in the KEGG BRITE dataset. And they pre-trained the CNN model on other non-lung cancer types to get more accurate prediction results [19]. Lyu et al. proposed to convert gene expression data into image for cancer type classification based on CNN. They padded genes into images, and the genes’ positions in the image are determined by their relative positions in the chromosome [28]. Sharma et al. proposed DeepInsight, a method for converting gene expression data into 2D images by the thought of clustering. They firstly compute the similarity of features to determine the position of genes in the feature matrix. Then they rearranged the features in the matrix by kPCA (kernel principal component analysis) or t-SNE (t-distributed stochastic neighbor embedding) [29]. Jha et al. made use of the gene expression data and clinical data to construct knowledge graphs. Then they used a deep graph convolutional neural network to predict the relapse in breast cancer [30]. Oh et al. proposed PathCNN, they creatively combined three kinds of omics data (gene expression data, copy number variation data, and DNA methylation data) into images. Then they used CNN to predict the long term survival of glioblastoma multiforme (GBM) and Grad-Cam was used for interpreting the prediction. [20]. Mohamed et al. introduced a bio-inspired convolutional neural network architecture that effectively utilized RNA-seq data for automatic breast cancer detection and classification. This innovative approach outperformed traditional methods, offering promising potential for improving breast cancer diagnosis [31].

### Model robustness and external validation

When we train a Machine Learning model, we often want it to learn the domain knowledge, and then naturally produce satisfactory prediction output. However, the truth is sometimes different. If only the internal validation paradigms such as cross-validation are used to test models’ performance, it is not enough to prove the domain knowledge has been captured by the models, therefore we cannot say such models are robust. Zech et al. implemented a medical imaging study, they observed that the CNN model they trained on the X-ray images was making predictions mostly depending on the word *portable* on the images. This word only indicated the type of the X-ray machine rather than the medical knowledge [32]. Sometimes the random selected gene signatures may even outperform carefully picked gene signatures filtered through rigorous statistical processes, on prediction accuracy. This phenomenon is called random signature superiority (RSS) [33]. Goh et al. proposed to leverage additional good analytical practices (GAPs) to evaluate the gene signatures with known sources of confounding genes in the gene expression data. Thus the tested models or gene signatures can be considered robust [34]. Ho et al. studied model validation in depth. They found that models with good internal validation performance sometimes could not capture the domain relevant features and had poor generalization performance. They proposed to use external validation to test whether a model is robust. And two kinds of external validation paradigms were designed, they were (1) convergent validation and (2) divergent validation [35].

## Data Availability

We use public datasets for the study in this paper. Here we provide the download links of the datasets we use: TCGA-LUAD: https://lce.biohpc.swmed.edu/lungcancer/datasetsearch.php?datasetid=60 TCGA-LUSC: https://lce.biohpc.swmed.edu/lungcancer/datasetsearch.php?datasetid=61 Oncosg_2020: https://www.cbioportal.org/study/summary?id=luad_oncosg_2020 TCGA-SKCM: https://www.cbioportal.org/study/summary?id=skcm_tcga Dfci_2015: https://www.cbioportal.org/study/summary?id=skcm_dfci_2015 Datasets with prefix ’GSE’: https://www.ncbi.nlm.nih.gov/geo/query/acc.cgi?acc=GSE2088https://www.ncbi.nlm.nih.gov/geo/query/acc.cgi?acc=GSE2514https://www.ncbi.nlm.nih.gov/geo/query/acc.cgi?acc=GSE5843https://www.ncbi.nlm.nih.gov/geo/query/acc.cgi?acc=GSE10072https://www.ncbi.nlm.nih.gov/geo/query/acc.cgi?acc=GSE11969https://www.ncbi.nlm.nih.gov/geo/query/acc.cgi?acc=GSE12667https://www.ncbi.nlm.nih.gov/geo/query/acc.cgi?acc=GSE13213https://www.ncbi.nlm.nih.gov/geo/query/acc.cgi?acc=GSE17475https://www.ncbi.nlm.nih.gov/geo/query/acc.cgi?acc=GSE20853https://www.ncbi.nlm.nih.gov/geo/query/acc.cgi?acc=GSE20875https://www.ncbi.nlm.nih.gov/geo/query/acc.cgi?acc=GSE26939https://www.ncbi.nlm.nih.gov/geo/query/acc.cgi?acc=GSE29016https://www.ncbi.nlm.nih.gov/geo/query/acc.cgi?acc=GSE31210https://www.ncbi.nlm.nih.gov/geo/query/acc.cgi?acc=GSE32863https://www.ncbi.nlm.nih.gov/geo/query/acc.cgi?acc=GSE41271https://www.ncbi.nlm.nih.gov/geo/query/acc.cgi?acc=GSE72094https://www.ncbi.nlm.nih.gov/geo/query/acc.cgi?acc=GSE68571https://www.ncbi.nlm.nih.gov/geo/query/acc.cgi?acc=GSE83227https://www.ncbi.nlm.nih.gov/geo/query/acc.cgi?acc=GSE68465https://www.ncbi.nlm.nih.gov/geo/query/acc.cgi?acc=GSE74777

## Code availability

The implementation of SurvConvMixer is now available on GitHub: https://github.com/PPDPQ/SurvConvMixer
